# OntoMate: a text-mining tool aiding curation at the Rat Genome Database

**DOI:** 10.1093/database/bau129

**Published:** 2015-01-25

**Authors:** Weisong Liu, Stanley J. F. Laulederkind, G. Thomas Hayman, Shur-Jen Wang, Rajni Nigam, Jennifer R. Smith, Jeff De Pons, Melinda R. Dwinell, Mary Shimoyama

**Affiliations:** ^1^Human and Molecular Genetics Center, Medical College of Wisconsin, ^2^Department of Quantitative Health Sciences, University of Massachusetts Medical School, ^3^Department of Physiology, Medical College of Wisconsin and ^4^Department of Surgery, Medical College of Wisconsin, 8701 Watertown Plank Rd, Milwaukee, WI 53226-3548, USA

## Abstract

The Rat Genome Database (RGD) is the premier repository of rat genomic, genetic and physiologic data. Converting data from free text in the scientific literature to a structured format is one of the main tasks of all model organism databases. RGD spends considerable effort manually curating gene, Quantitative Trait Locus (QTL) and strain information. The rapidly growing volume of biomedical literature and the active research in the biological natural language processing (bioNLP) community have given RGD the impetus to adopt text-mining tools to improve curation efficiency. Recently, RGD has initiated a project to use OntoMate, an ontology-driven, concept-based literature search engine developed at RGD, as a replacement for the PubMed (http://www.ncbi.nlm.nih.gov/pubmed) search engine in the gene curation workflow. OntoMate tags abstracts with gene names, gene mutations, organism name and most of the 16 ontologies/vocabularies used at RGD. All terms/ entities tagged to an abstract are listed with the abstract in the search results. All listed terms are linked both to data entry boxes and a term browser in the curation tool. OntoMate also provides user-activated filters for species, date and other parameters relevant to the literature search. Using the system for literature search and import has streamlined the process compared to using PubMed. The system was built with a scalable and open architecture, including features specifically designed to accelerate the RGD gene curation process. With the use of bioNLP tools, RGD has added more automation to its curation workflow.

**Database URL:** http://rgd.mcw.edu

## Introduction

The Rat Genome Database (RGD, http://rgd.mcw.edu) has always looked for ways to improve curation efficiency by making use of software tools. From 2006 to 2009, the bioinformatics developers at RGD created a tool suite (1) to assist RGD’s curation process. The tools improved a process that originally had been based on spreadsheet data entry. The ontology annotation creation and editing tool serves as a data entry interface for the curation database. Until recently, RGD biocurators relied on literature searches using PubMed’s interface (2) to locate articles for curation. To improve the workflow, the curators wanted a search engine which could interface with the gene curation tool.

BioCreative: Critical Assessment of Information Extraction in Biology is a community-wide effort to promote creation and improvement of text-mining tools and solutions for biocuration by organizing common evaluation tasks. For the BioCreative 2012 (3–5) and 2013 (6,7) workshops several RGD curators tested various text-mining tools. Among them, RGD curators found PubTator (http://www.ncbi.nlm.nih.gov/CBBresearch/Lu/Demo/PubTator/index.cgi?user=User422218159) most similar to what was desired for their gene curation workflow, because RGD gene curators annotate disease, phenotype, pathway and Gene Ontology (GO) data from the biomedical literature. All those different data types need to be curatable through the same interface simultaneously.

PubTator (8) is a web-based curation-assisting biomedical search engine. It utilizes biological natural language processing (bioNLP) tools to annotate and index information useful for biocuration, such as genes, disease terms and species. Much more biocurator-friendly than PubMed, PubTator lacks some features that were needed for RGD curation. For example, articles in the query result can only be sorted by publication date, not by relevance. Most importantly, PubTator is not flexible enough to be directly integrated into RGD’s curation workflow. After researching different solutions, RGD decided to create and integrate OntoMate, into its gene curation workflow.

## OntoMate

OntoMate is an ontology-driven, concept-based literature search engine. The original goal of the project was to utilize text-mining tools to partially automate the development of ontologies used in PhenoMiner (9–12). PhenoMiner is a repository of rat phenotype data. It utilizes four ontologies developed at RGD: the rat strain ontology, the clinical measurement ontology, the measurement method ontology and the experimental condition ontology. The basic requirement of OntoMate was to be able to tag ontology terms in free text articles (MEDLINE abstracts) and generate term-article statistics. The first requirement was a typical information extraction tool and the second was an information retrieval application. After comparing different solutions to the problems, we decided to build an in-house system. Although there are plenty of information extraction and information retrieval tools, few of them can address the two needs that RGD has simultaneously:

1. Ontologies are constantly changing. This is very different from most tagging systems’ assumptions: execute once and save the results. Our plan was to apply the tagging system upon each new release of an ontology. To tag terms of these ontologies, a very customizable tagging system was required.
2. We needed to process all MEDLINE abstracts with the tagging system. The performance of a tool such as the National Center for Biomedical Ontology (NCBO) Annotator (13) would not allow us to annotate the whole document set as often as necessary. Besides the basic requirement of tagging, we also wanted to extend the system to assist curators in gene curation and other text-mining tasks.

In designing the system, we set a few more requirements:

1. The system should have high performance that allows processing a large volume of text files in a relatively short time.
2. The system should be scalable. When needed, it should not be difficult to add more servers to the system to expand processing and storage capacity.
3. The system should be robust. Single-server failure should not cause data loss or system failure.
4. The system should be open. It should not be cumbersome to integrate third party tools into the system.
5. The system should use open source software as much as possible to keep costs low.

These requirements were not just for the tagging task but also for more text-mining tasks over time. Since Java is the first choice for most bioNLP developers, it was used throughout the system development. As the project progressed, we have not only fulfilled the original requirements but also have successfully integrated OntoMate into RGD’s gene curation workflow.

## System architecture and implementation

**Figure 1.**
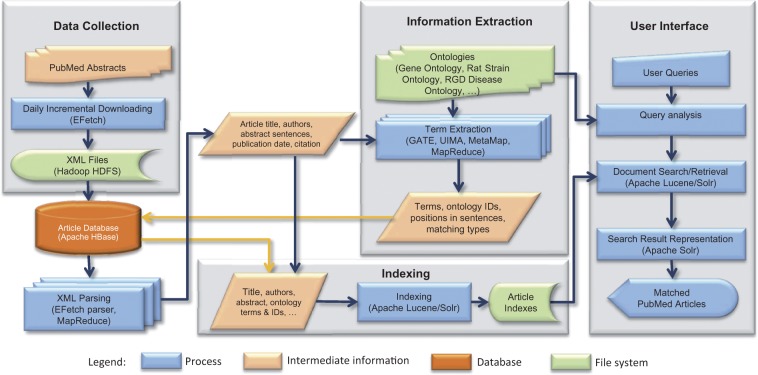
OntoMate system architecture. The basic system consists of data collection, article database, information extraction and information retrieval (indexing and user interface). User interface can be adapted for different applications.

OntoMate consists of four main components: data collection, article database, information extraction and information retrieval (Figure 1).

### Data collection

Since almost all of RGD’s gene curation has relied on the biomedical literature search engine at PubMed, OntoMate currently uses PubMed as the sole data source. At present, OntoMate processes the title, the abstract and the Medical subject headings terms of each article. Full-text processing is planned for the future. A pipeline was built using NCBI’s E-utilities Web Service (SOAP-Simple Object Access Protocol) (http://www.ncbi.nlm.nih.gov/books/NBK25500/) to retrieve data from the PubMed database. The pipeline uses the Apache Axis2 for Java programming interface, because it includes an Extensible Markup Language (XML) parser that can access any field in the XML files retrieved from PubMed. Initially, the pipeline retrieved all records in the PubMed database. This process originally took several weeks to finish. After the initial download was complete, the pipeline has run every night to only add new articles made available during the previous day. Finally, the pipeline parses all the downloaded XML files and loads the articles into the article database.

### Article database

This is a crucial component regarding system performance, because it is involved in every operation before the article index is created. We have evaluated several database systems for storing articles along with annotations and other related information. MySQL (http://www.mysql.com/) was determined to be too slow, not adequately scalable and too costly for storing many millions of articles with billions of tagging annotations attached.

NoSQL (http://nosql-database.org/) is a next-generation database that addresses some of our problems with SQL. NoSQL is non-relational, distributed, open-resource and horizontally scalable. By definition, NoSQL should serve our application much better than a MySQL database. We have tested two NoSQL solutions: Apache CouchDB (http://couchdb.apache.org/) and Apache Hadoop/HBase (http://hadoop.apache.org/) (14). We chose Hadoop/HBase, because it provided better performance and flexibility than CouchDB could offer.

Apache HBase is an open-source, distributed, versioned and column-oriented database. HBase runs over Hadoop distributed file system (HDFS) (http://hadoop.apache.org/docs/r1.2.1/hdfs_design.html). This enables Hadoop Map/Reduce (15) jobs to read database records from a local server; hence, system throughput is not bounded by network bandwidth. Records in tables are stored as key-value pairs. We used reversed (for load balancing) PubMed identification number (PMID) as the key. Each record can have millions of columns that can be written or read individually. Columns in HBase are grouped into column families. We decided to store the article in XML format in one column. Each time we need to access an attribute in an article, the complete XML data is read from the database and parsed. In our current setup, it takes 34 min to read and parse all the articles in XML, where <2 min is spent on parsing.

Columns of HBase records are versioned by having a timestamp on each stored value. The user can define the number of versions to keep. When a new value is received, the database will automatically compare its timestamp with existing values and only keep the most recent ones. This feature is very attractive to us as we do not have to delete the existing annotations when re-running the taggers. The timestamps also facilitate tracking of article changes in PubMed. We built a small Hadoop cluster using eight Dell servers. It only requires 1.5 h to load all 23 million articles into HBase, compared with 71 h with a single MySQL server. Scanning through all articles takes only 24 min.

### Information extraction

To make the best use of existing tools developed by the NLP community, we decided to use the popular NLP framework GATE (General Architecture for Text Engineering) (16). We built a dictionary-based ontology term-tagging pipeline using GATE plugins. The pipeline comprised two ANNIE (A Nearly-New Information Extraction System) Gazetteer plugins: one for case- sensitive terms and one for case-insensitive terms. ANNIE is a set of basic information extraction libraries.

We exported ontology terms from the RGD database and stemmed them using Snowball Stemmer to build term dictionaries. Article texts are also stemmed before running through the pipeline. Stemming is the process of reducing a word to its root form. For example, ‘creatures’ becomes ‘creatur’ after stemming. All annotations of an article from the pipeline are stored in HBase as one column. In our tests, the results generated by our pipeline are very close to those of the NCBO Annotator. Our ontology tagging pipeline tags terms not only from the five ontologies used by PhenoMiner but also from most of the other 11 ontologies used by RGD. Besides the ontology tagging pipeline, we built a gene-tagging pipeline, an organism-tagging pipeline, a mutation-tagging pipeline and a part of speech tagging pipeline using GATE plugins.

Unlike most of the information extraction systems for biocuration, gene annotations are not normalized in OntoMate. One of the reasons for this is because it is very difficult to precisely map gene mentions to organism specific gene identifiers that will be used in making annotations. Incorrect mappings could cause loss in recall in the information retrieval step. Another reason is because RGD’s gene-disease curation process always starts from a given gene identifier. Mapping from a gene identifier to a gene mention is relatively straightforward. Mismatch is less likely to happen if organism constraint is ignored. The ABNER (A Biomedical Named Entity Recognizer) (17) gene annotator only marks text segments that are more likely to be gene-related text. Searching for genes in articles is still keyword based, which relies on the query-expansion function that is discussed below in the ‘Query submission and analysis’ section.

### Information retrieval

HBase is a good solution in our application to serve as an article/annotation store, but it lacks a convenient means to query the data. We decided to index the data in HBase for query using Apache Solr (http://lucene.apache.org/solr/). Solr is based on the Apache Lucene project (http://lucene.apache.org/core/index.html), which is a high-performance, full-featured text search engine library. Solr makes it easy to use Lucene by offering features like hit highlighting, faceted search, rich document handling and distributed indexing. Faceted search can generate the value distribution of a given field, which is ideal for obtaining term-document statistics. High-performance full-text search also makes it possible for us to build our own literature search engine for curation at RGD. Solr also provides REST (REpresentational State Transfer)-like HTTP/XML and JavaScript Object Notation (JSON) Application Programming Interfaces (APIs) as convenient programming interfaces for submitting queries and retrieving results. With the distributed indexing feature, it takes less than 5 h to index all 23 million articles and their annotations in our Hadoop cluster.

## Integrating OntoMate into the RGD gene curation workflow

**Figure 2.**
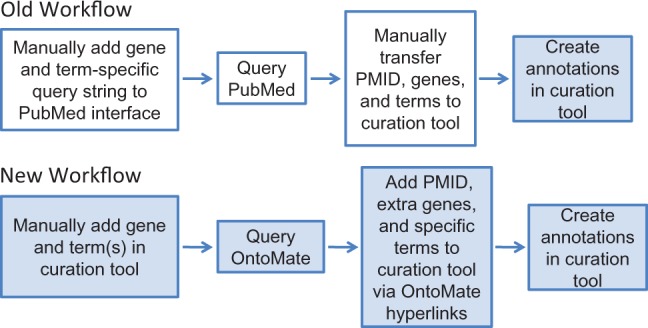
RGD’s old and new workflows for manual gene curation. White boxes represent tasks involving the PubMed interface and colored boxes represent processes done in the RGD curation tool interface. The new workflow has reduced the processes of the old workflow from two interfaces to one interface.

Gene curation at RGD is driven by disease-associated, pathway-associated and QTL-related projects. This involves curating disease terms, pathway terms and GO terms to the genes involved. The integration of OntoMate to the RGD gene curation workflow addresses this range of annotations. The old and current gene curation workflow at RGD is shown in Figure 2. OntoMate has merged a search function, previously provided by PubMed, with the RGD curation tool, such that the entire process of literature search, title/abstract curation and annotation is consolidated into one interface instead of two.

Table 1 lists query strings for a gene-disease search in PubMed compared with a gene-disease search in the OntoMate/curation tool interface. By using gene IDs and ontology term IDs, OntoMate allows the curator to minimize the manual search input while getting nearly the exact same results as the PubMed search. OntoMate relies on a gene and its orthologs to supply name, symbol and synonyms based on the gene’s RGD ID supplied in the curation tool. For gene-disease curation, RGD curators are only interested in papers related to rat, human or mouse, so the default search parameters for the three species are automatically appended to the query string. By using the strength of the ontology hierarchy (simultaneously searching for child terms of any parent term entered in the search box), OntoMate relieves the burden of entering many search terms to get results for a complex search.

**Table 1.** Comparison of term strings and gene query strings between PubMed and OntoMate searches

Disease category	PubMed searches (manually constructed)		OntoMate searches (automatically generated based on the input RGD disease ontology ID and RGD gene ID)	
	Term query string	Sample gene query string	Term query string	Sample gene query string
Kidney diseases	(kidney or renal or urethral or ureteral or urinary or bladder) and (disease or injury or disorder or insufficiency or obstruction or polycystic or cyst or failure or stones)	(ADSF or RSTN or XCP1 or FIZZ3 or retn or resistin)	‘Kidney Diseases’ (RDO:0000692)	‘Retn’ (RGD:628781)

These searches involved a single gene for the recent Renal Disease Portal curation at RGD. The OntoMate search is based on RGD gene IDs and ontology term IDs.

### OntoMate user interface for curation

**Figure 3.**
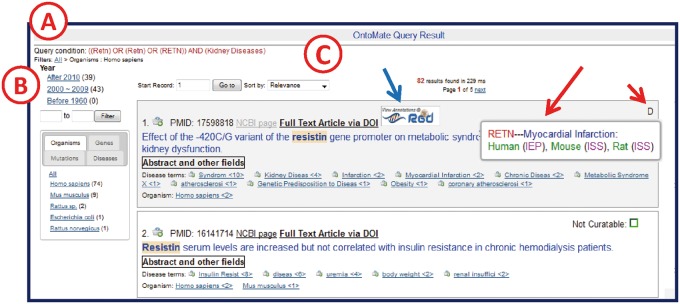
OntoMate query results page. (**A**) ‘Query Condition’ displays the string of objects and terms used in the query. (**B**) The filter section allows users to adjust the results according to publication chronology or object/term refinements. The tabs display hyperlinked subsets of result categories. Any link may be selected to restrict or expand the selected results. A ‘filter path’ appears below the Query Condition to show the user what filters have been applied. (**C**) The search results are sorted by relevance by default, but can also be sorted by publication date or PMID. If the reference is in RGD already, an RGD logo appears (blue arrow) above the title. If there are any GO or disease vocabulary annotations from this reference, an aspect initial appears in the upper right corner of the reference entry (short red arrow, D = disease). By ‘mousing over’ the aspect letter, a pop-up appears to show what annotation(s) has been made (long red arrow).

OntoMate’s query result user interface specially designed for RGD curation is shown in Figure 3. The user interface comprises three zones: the query condition zone, the filters zone and the article list zone. The query condition zone shows the conceptual query condition. The RGD gene IDs and term accession IDs from the actual query condition sent from the curation tool are converted to gene symbols and ontology terms, respectively, before rendering. Concepts are connected by Boolean operators that are used in the actual Solr query string.

In the *filters zone* clicking a hyperlink in the filter list will apply a filter to the query result to only include articles that have that concept or are in the publication date range. Multiple filters can be combined. The ‘filter path’ shows what filters have been applied to the result set (Figure 3). Users can remove filters by clicking an upper node in the path. The number after each filter hyperlink indicates how many articles will show if the filter is applied. Concepts are sorted by these numbers in descending order.

**Figure 4.**
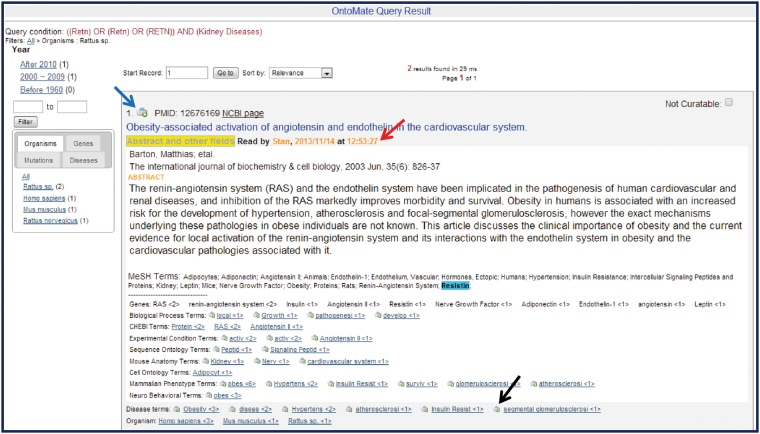
Sample OntoMate abstract entry. The abstract has been opened by clicking on the ‘Abstract and other fields’ button underneath the title. A ‘Read by’ (red arrow) note shows that the abstract has been accessed by another user sometime before. The abstract can be entered into the main RGD database and into the curation tool interface by clicking on the bucket icon (blue arrow) above the title. Any of the hyperlinked terms can be placed in the curation tool term bucket by clicking the bucket icon (example at black arrow) to the left of the appropriate term. Terms may also be displayed in the term browser in the curation tool interface by clicking the hyperlinked term. Corresponding terms in the abstract are highlighted if the user ‘mouses over’ any of the terms listed below the abstract.

The *article list zone* by default ranks the articles by relevance. Given a query, Lucene calculates the relevance of each article that matches the query using a similarity scoring formula (18). Users can change to two other sorting conditions, by publication date or by PMID, from the ‘Sort by’ drop-down text box. Each reference entry has various links to both the RGD curation interface and to external sources for full paper access [PubMed Central (http://www.ncbi.nlm.nih.gov/pmc/) or publisher web sites]. If the reference is already in RGD, clicking the RGD logo above the title opens the article’s reference report page at RGD. For disease annotations and GO annotations, the user interface will display letters of the corresponding aspects in the upper right corner of the article entry. Mousing over an aspect letter reveals annotation details (Figure 3). If the abstract of the article has been opened in OntoMate, the tool will show a ‘Read by’ indicator with the curator’s name and timestamp (Figure 4). This feature, along with the previous annotation indicators, prevents articles from being unnecessarily re-read, which is not possible within the PubMed results interface. If the article has no annotations in RGD and the title appears curatable, the curator can click the ‘Abstract and other fields’ button to reveal the abstract, the citation details and lists of any ontology/ vocabulary terms with which the abstract has been automatically annotated/tagged (Figure 4).

The abstract-opening action will be immediately logged so that the information can be used by the ‘Read by’ indicator. The abstract of the article displays on the same page so that the curator does not need to go back and forth between the article list page and an abstract page as in PubMed. The curator can hide the details by clicking the button again. Matched gene names/symbols are automatically highlighted in the title and abstract. The curator can mouse over an ontology term to manually highlight it in the body of the abstract. Each annotated term is a link to the term in the ontology browser in the curation tool interface. Terms are followed by number of their occurrences and are sorted by this number in descending order. When a curator decides to make annotations from the article, he/she can directly import the article to the curation tool by clicking the bucket icon to the left of the PMID on the query results page. This one click replaces the ‘copy/paste/click to import’ procedure that curators used when curating from PubMed. The curator can also add an ontology/vocabulary term to the curation tool directly from the list under the abstract. This is also achieved by clicking the bucket icon to the left of any term. As soon as the disease or GO term associations are entered into the RGD database, the OntoMate query result page will automatically display the new annotations. The action of importing articles, adding terms to curation and accessing full-text articles are all logged for analysis of curation work. Logs of accessing full-text articles can be used to indicate if the abstract was not sufficient for making an annotation.

## Summary

As the initial effort to integrate text-mining tools into RGD’s curation workflow, we created an interface between OntoMate and RGD’s curation tool, so that OntoMate could replace PubMed as the literature search engine for gene curation. The average number of papers curated per curator per hour has increased from 2.10 to 2.83 after switching to OntoMate. Curators do not need to spend time constructing query strings for genes and diseases. This amounts to about 5 min in time savings per gene/ontology term search. Re-reading previously curated papers now can be easily prevented. The hyperlinked PMIDs, gene names and ontology/vocabulary terms in OntoMate create a shortcut to the RGD curation tool that eliminates a lot of copy/pasting, typing and term searching that was previously needed when curating the literature from PubMed. This accounts for about 10–15 sec in time-savings per ontology term annotation and 5–10 s per PMID loaded into the curation tool/database. Regarding precision and recall of query results, the curators have not found any difference between OntoMate and PubMed results.

The most attractive potential of using OntoMate in RGD curation is that it is open and fully customizable. Logging of sentence-level user interactions will be added. We will use machine learning techniques to build models to decide which articles are more likely suitable for curation or even automatically generate gene-term associations. The log data we collect of user actions will be very useful in building these kinds of models. We will add more pipelines to extract information that is useful to curation or machine learning, such as syntax information, dependencies, key words/sentences and events. Using the system infrastructure of OntoMate, we will annotate all full-text articles available to us and make the information directly accessible in the OntoMate curation user interface. More user-interactive features will be added in the future.

## Funding

This work was supported by the National Heart, Lung and Blood Institute on behalf of the National Institutes of Health (HL064541 and HL094271). Funding for open access charge: National Heart, Lung and Blood Institute on behalf of the National Institutes of Health (HL64541).

*Conflict of interest*. None declared.
